# Supplementary figures and images for: A global view of drug-therapy interactions
Source: BMC Pharmacol. 2008 Mar 4;8:5. doi: 10.1186/1471-2210-8-5 (PMC2294115; doi:10.1186/1471-2210-8-5)

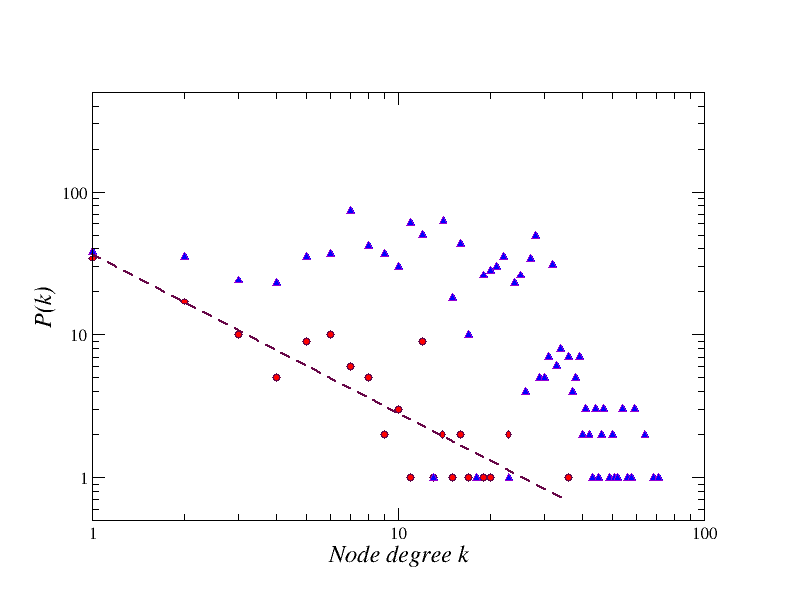

Supplement: Additional file 2 — Comparison between the degree distributions of the therapy (circles) and drug (triangles) projections at level 3 of the ATC classification. [file 1471-2210-8-5-S2.png]

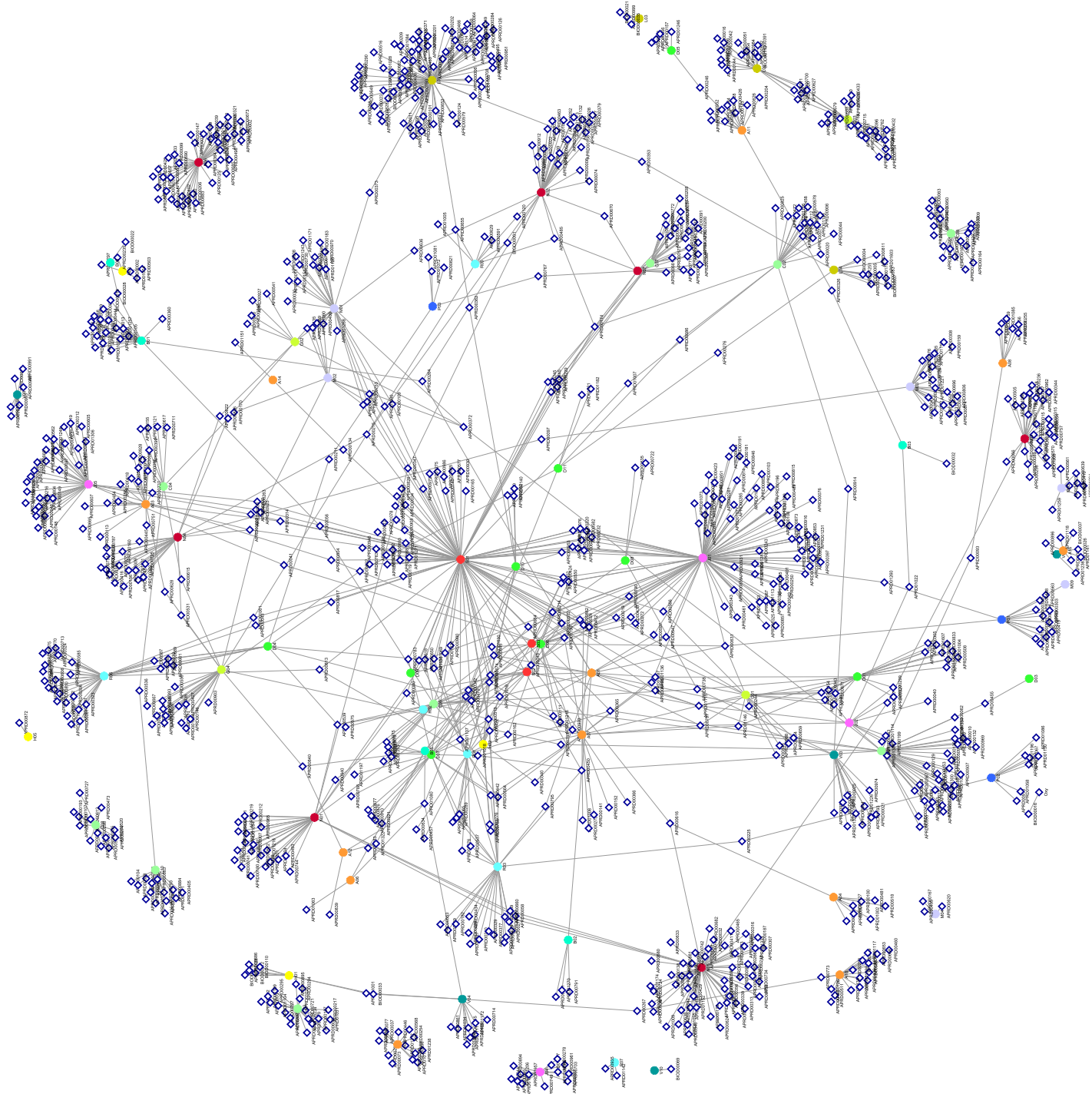

Supplement: Additional file 3 — Full bipartite drug-therapy network at level 2 of the ATC classification. Drugs are represented by dark blue empty diamonds, therapies are represented by circles and are colored following the same code as in Figure 1 shown in the main text. [file 1471-2210-8-5-S3.pdf]

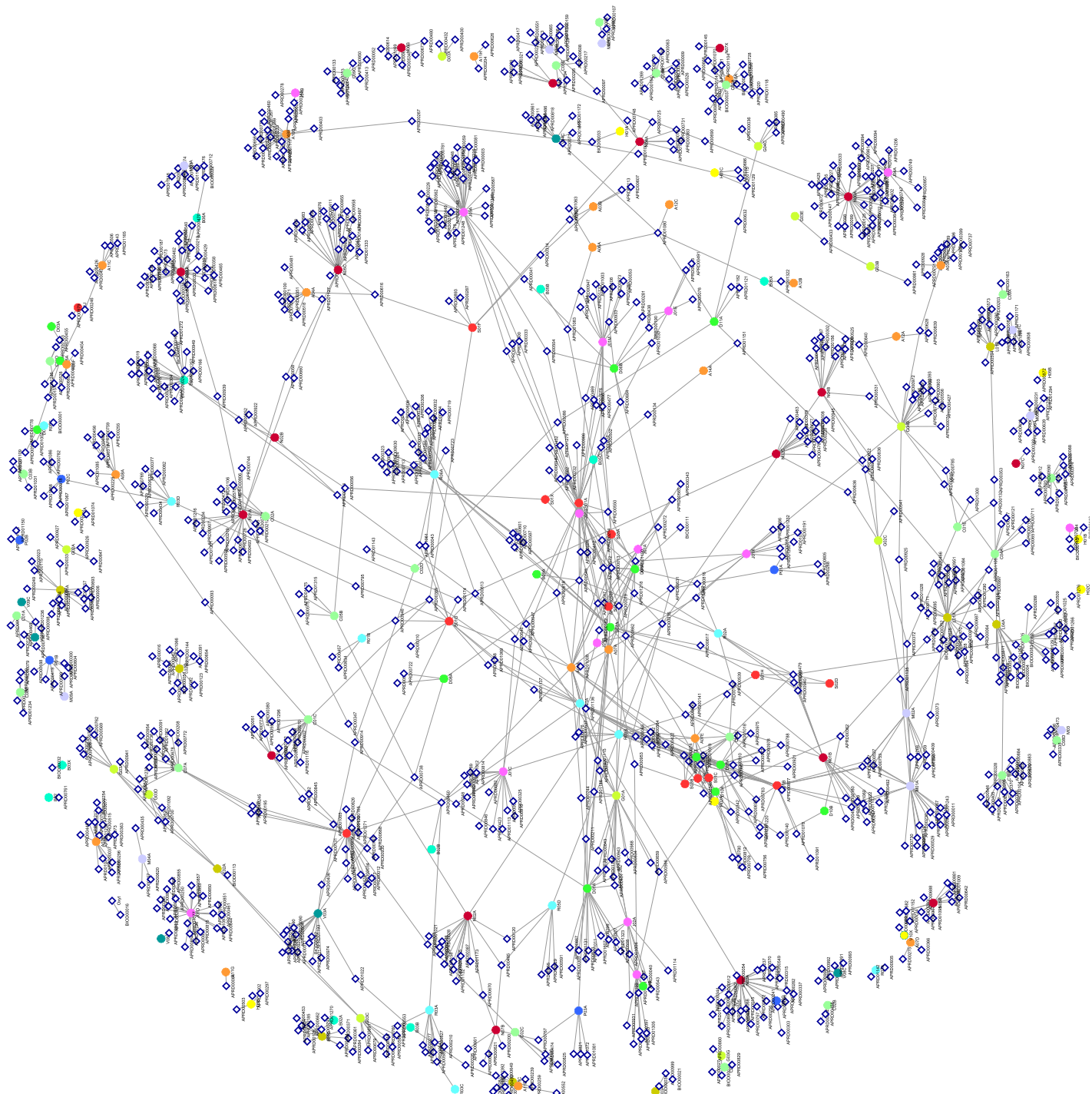

Supplement: Additional file 5 — Full bipartite drug-therapy network at level 3 of the ATC classification. [file 1471-2210-8-5-S5.pdf]

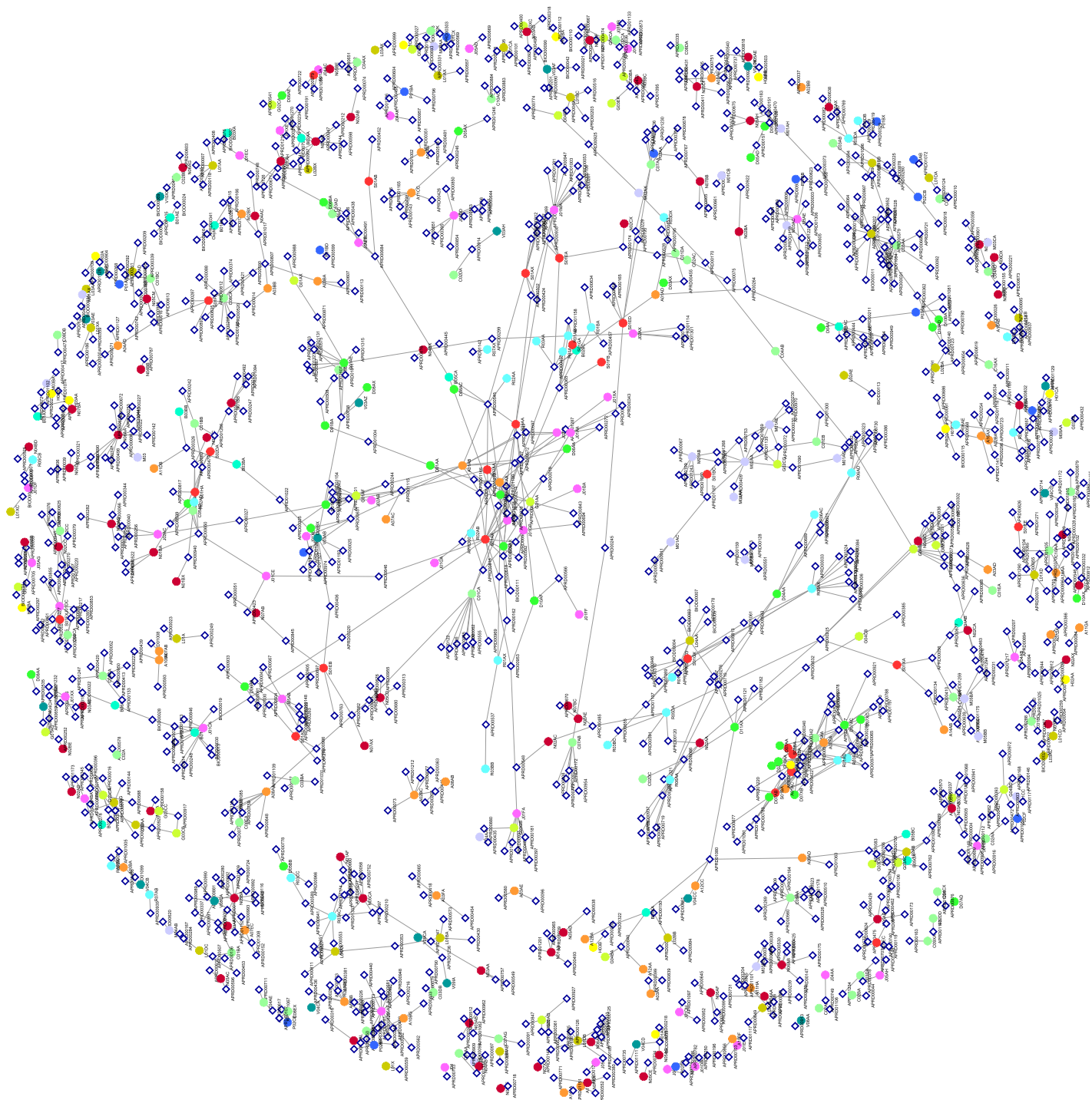

Supplement: Additional file 7 — Full bipartite drug-therapy network at level 4 of the ATC classification. [file 1471-2210-8-5-S7.pdf]

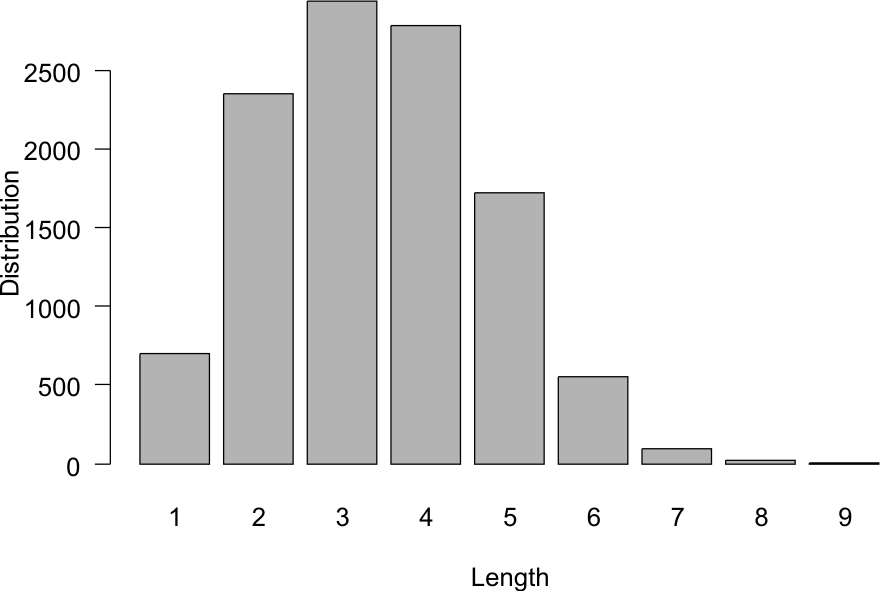

Supplement: Additional file 9 — Distribution of shortest path lengths in level 3 of the therapy network. [file 1471-2210-8-5-S9.png]
